# Electrochemical Sensors for Antibiotic Detection: A Focused Review with a Brief Overview of Commercial Technologies

**DOI:** 10.3390/s24175576

**Published:** 2024-08-28

**Authors:** Margaux Frigoli, Mikolaj P. Krupa, Geert Hooyberghs, Joseph W. Lowdon, Thomas J. Cleij, Hanne Diliën, Kasper Eersels, Bart van Grinsven

**Affiliations:** Sensor Engineering Department, Faculty of Science and Engineering, Maastricht University, P.O. Box 616, 6200 MD Maastricht, The Netherlands; mp.krupa@student.maastrichtuniversity.nl (M.P.K.); geert.hooyberghs@maastrichtuniversity.nl (G.H.); joe.lowdon@maastrichtuniversity.nl (J.W.L.); thomas.cleij@maastrichtuniversity.nl (T.J.C.); hanne.dilien@maastrichtuniversity.nl (H.D.); kasper.eersels@maastrichtuniversity.nl (K.E.); bart.vangrinsven@maastrichtuniversity.nl (B.v.G.)

**Keywords:** antibiotics, antibiotic resistance, antimicrobial resistance, analytical techniques, biosensors, optical biosensors, electrochemical biosensors, antibiotic residue

## Abstract

Antimicrobial resistance (AMR) poses a significant threat to global health, powered by pathogens that become increasingly proficient at withstanding antibiotic treatments. This review introduces the factors contributing to antimicrobial resistance (AMR), highlighting the presence of antibiotics in different environmental and biological matrices as a significant contributor to the resistance. It emphasizes the urgent need for robust and effective detection methods to identify these substances and mitigate their impact on AMR. Traditional techniques, such as liquid chromatography-mass spectrometry (LC-MS) and immunoassays, are discussed alongside their limitations. The review underscores the emerging role of biosensors as promising alternatives for antibiotic detection, with a particular focus on electrochemical biosensors. Therefore, the manuscript extensively explores the principles and various types of electrochemical biosensors, elucidating their advantages, including high sensitivity, rapid response, and potential for point-of-care applications. Moreover, the manuscript investigates recent advances in materials used to fabricate electrochemical platforms for antibiotic detection, such as aptamers and molecularly imprinted polymers, highlighting their role in enhancing sensor performance and selectivity. This review culminates with an evaluation and summary of commercially available and spin-off sensors for antibiotic detection, emphasizing their versatility and portability. By explaining the landscape, role, and future outlook of electrochemical biosensors in antibiotic detection, this review provides insights into the ongoing efforts to combat the escalating threat of AMR effectively.

## 1. Introduction

Pathogens are dangerous classes of microorganisms, including bacteria, viruses, fungi, or parasites, that can cause different types of diseases in their host organisms [1]. These microbes have the ability to infect humans, animals, and plants, succeeding in multiplying within the tissues of the host, thus potentially leading to infections and various health problems. While prevention methods such as proper hand hygiene and vaccination are still the most cost-effective and sustainable approaches to stopping the spread of bacterial infections, the discovery of antibiotics was also crucial in controlling and treating them [2]. However, pathogens are generating increasingly more issues for the environment, healthcare, and food safety due to their ability to develop adaptivity to diverse conditions, including resistance to specific drugs used to eradicate them. Antimicrobial resistance (AMR) occurs when microorganisms, including bacteria, evolve mechanisms to evade the effects of drugs designed to inhibit their growth or kill them. This resistance can arise not only from the evolution of new resistant strains but also from the expansion of naturally resistant populations or through intrinsic resistance mechanisms [3]. In addition, it is important to note that some antibiotics act by inhibiting bacterial growth (bacteriostatic) rather than directly killing bacteria (bactericidal) [4]. AMR, or antimicrobial resistance, refers to the ability of various microbes—including bacteria, viruses, fungi, and parasites—to withstand the effects of a broad range of antimicrobial agents. According to the WHO, antibiotics are a specific subset of antimicrobial agents that target bacteria. Thus, antibiotic resistance (AR or ABR) specifically pertains to bacterial resistance to these drugs. It is important to note that in many scientific publications, the terms AMR and AR are often used interchangeably when discussing bacterial resistance [5]. The problem of antimicrobial and antibiotic resistance is becoming increasingly overwhelming. According to the Centers for Disease Control and Prevention (CDC), the US alone experiences over 2.8 million AMR-related infections each year, leading to more than 35,000 deaths. These infections include both community-acquired and nosocomial infections, which differ in their origins and settings. Community-acquired infections occur outside of healthcare settings, while nosocomial infections are acquired during hospital stays. Both types are significantly impacted by AMR, highlighting the widespread nature of the problem [6,7]. This acquired resistance creates infections that are challenging or even impossible to treat, which further increases the spreading of diseases, severe illnesses, and death. Antimicrobial-resistant pathogens cause health complications such as pneumonia and liver abscesses, as well as several infections ranging from skin and soft tissue infections to urinary tract infections [8]. The six main nosocomial antimicrobial-resistant pathogens that are primarily to blame for these health complications have been coined under the acronym ESKAPE and they include *Enterococcus faecium*, *Staphylococcus aureus*, *Klebsiella pneumoniae*, *Acinetobacter baumannii*, *Pseudomonas aeruginosa*, and *Enterobacter species* [9].

The development of AMR occurs through two mechanisms. Firstly, gene inheritance occurs via mobile genetic elements through horizontal gene transfer (HGT), allowing antibiotic resistance to be transferred among different species. There are three types of HGT: transformation, conjugation, and transduction [10]. Transformation occurs when bacteria take up DNA from their environment, including exogenous DNA that encodes antibiotic-resistant genes, which may have been released by dead bacteria. This DNA is then incorporated into the host bacterium with the help of specific competence factors. Conjugation is the process of direct gene transfer between bacteria via a specialized conjugation tube or pilus. Finally, transduction is the action of bacteriophages transferring genes from one cell to another, including AMR genes to bacterial cells. After gene transfer, pathogens continue to adapt through various mechanisms, including genetic mutations and the acquisition of new resistance genes. In the case of antibiotic resistance, the overuse of antibiotics employs natural selection pressure, which promotes the proliferation of resistant strains [11]. In addition, random genetic mutations can take place, which result in variations in translated proteins, or increases/decreases in gene expression, that allow for antibiotic resistance [12]. When antibiotics are administered to humans or animals, they kill or prevent the growth of the target bacteria. Inevitably, only bacteria that did not develop resistance are killed, leaving behind those with natural or acquired resistance. This process is a form of natural selection at the microbial level and those with antibiotic-resistant genes can pass on these advantageous traits to their offspring [13]. Over time, this can lead to the proliferation of antibiotic-resistant bacterial strains, as their resistance genes are inherited by subsequent generations. However, it is important to note that resistance genes may impose a fitness cost on bacteria, with the consequence that without antibiotic selection pressure, the frequency of these resistant strains may decrease [14]. The main mechanism of AR may be separated into four principal strategies employed by bacteria: (a) limiting the uptake of a drug, namely reducing the permeability of the cell membranes to prevent the drug from entering the cell; (b) modifying a drug target, a genetic ability that bacteria can use to modify the molecules or the receptors targeted by the antibiotics, (c) inactivating a drug, which usually happens through the production of specific enzymes that can chemically modify or degrade the antibiotic, and lastly, (d) active drug efflux, namely the ability of bacteria to actively transport the drug outside the cell before it reaches the target [15]. Notably, Gram-negative bacteria can use all four processes, whereas Gram-positive bacteria do not have the capacity for certain drug efflux mechanisms or the ability to limit the uptake of the drug due to lacking a lipopolysaccharide outer membrane, making Gram-negative bacteria generally more difficult to fight [16].

Numerous causes may be attributed to the development of AMR and AR, including antibiotic misuse, inaccurate prescribing, excessive use in agriculture and livestock, inefficient wastewater treatment, and lack of novel antibiotics [17]. The excessive use of antibiotics in agriculture results in the transfer of antibiotic-resistant bacteria to humans through products such as cereals, meat, eggs, milk, vegetables, fruit, and even water [18,19]. In addition, the redundant use of antibiotics in livestock leads to detrimental effects on the environment. For example, up to 90% of antibiotics administered to livestock are excreted through urine and feces and are then spread to local groundwater [20]. Antibiotic misuse in humans is another prevalent cause of AR, with antibiotics being administered too indiscriminately [21]. The importance of the detection of antibiotics and AMR microorganisms has been highlighted by the “One Health” approach adopted by the WHO, the World Organization for Animal Health (WOAH—formerly OIE), the United Nations Food and Agriculture Organization (FAO), and the United Nations Environment Programme (UNEP). The “One Health“ approach is built on the concept that human health is intricately connected to those of other animals and the environment that they inhabit [22].

Besides environmental applications, antibiotic detection is important in clinical development [23,24]. In fact, a key step during the development of novel antibiotics is translational Pharmacokinetic/Pharmacodynamic (PK/PD) studies, which require accurate antibiotic concentration measurements to predict the efficacy of antibiotics in animal models and humans [25,26]. During patient treatment, antibiotic detection plays a crucial role in Therapeutic Drug Monitoring (TDM) to allow for personalized medicine [27]. In 2019, the WHO published a list of drugs that require TDM, which contained all major classes of antibiotics [28]. Previously, this concern was addressed through pharmaceutical development, with new antibiotics being synthesized to overcome the issue of resistance. In 2021, an annual WHO analysis identified 27 new antibiotics in clinical development targeting priority pathogens [28]. Furthermore, between 2017 and 2022, 12 novel antibiotics were approved, with 10 of these belonging to antibiotic classes that are particularly susceptible to resistance [29]. Due to this continuous drive towards the development of new antibiotics, the development of fast, accessible, and ready-to-use antibiotic detection methods has acquired paramount importance for both industry and academia, leading to the evolution of technologies and novel publications presenting a wide variety of alternatives to fight the increasing number of problems generated by AR and AMR [30]. In addition, many countries have adopted strict maximum residue limits for antibiotics in water and food to limit the effect of residual antibiotics, resulting in the need for sensitive detection techniques for antibiotics [31].

Currently, the gold-standard analytical methods for quantification of antibiotics in biological, food, and environmental samples are liquid chromatography (LC), typically paired with mass spectrometry (MS), gas chromatography (GC), and capillary electrophoresis (CE) [32]. The most prevailing LC technique is high-performance liquid chromatography (HPLC), which is commonly employed due to its sensitivity as well as specificity for identifying antibiotics such as β-lactam compounds [33], namely one of the most prescribed antibiotic classes that include penicillins, carbapenems, and monobactams [34]. β-lactams’ mechanism of action includes interruption of cell wall formation due to covalent binding to penicillin-based proteins (PBPs), enzymes involved in the vital cross-linking of peptidoglycan in both Gram-negative and Gram-positive bacteria [35]. The LoDs for the detection of β-lactams in serum and interstitial fluids were found to be 0.003 mg/L [36].

On the other hand, an alternative to the gold-standard technologies is represented by immunoassays such as enzyme-linked immunosorbent assays (ELISAs) and immunochromatographic assays (ICAs). These technologies are common examples of screening techniques that utilize antibodies for screening antibiotic residues [37]. They enable the detection of antibiotics through the linking of the analyte to a specific antibody [38]. Since these reactions are stoichiometric, it is possible to determine the concentration of a given antibiotic based on the determination of either free or bound antigens [39]. ELISAs have high sensitivity, detects multiple samples at once, and allows for the screening of many small-volume samples [40,41,42]. Immunoassays may be characterized by low limits of detection (LoDs), as well as high specificity and sensitivity [43]. An example is the indirect competitive chemiluminescence enzyme immunoassay (IC-CLEIA) developed for the detection of chloramphenicol (CAP) residues in shrimp [43]. It was found that LoDs for the detection of various antibiotics ranged from 0.02 ng/mL to 0.5 ng/mL [44,45].

The methods for antibiotic detection presented above, although currently accepted in the industry, present numerous limitations. MS techniques require steep initial investments in equipment, as well as solid infrastructure [46]. In addition, LC-MS can struggle to produce precise quantitative results, particularly at lower concentrations of the analyte, which increases the likelihood of false-negative results [46]. In contrast, lateral flow immunoassays are designed to be more straightforward and rapid, but they typically require pre-treatment to minimize interference that could cause false positives. Such pre-treatments might include sample dilution, purification, the use of blocking agents, and buffer optimization [47]. While these steps are crucial for reducing non-specific interactions and improving assay accuracy, they can also delay the time of detection. Despite these challenges, lateral flow immunoassays have proven effective in detecting different analytes in point-of-need testing, as demonstrated, for instance, for COVID-19 [48]. These limitations impact the spread of AR as the ubiquity of antibiotics throughout the environment may not be addressed quickly enough. As an emerging alternative, biosensors have the potential to address these limitations by presenting extremely high sensitivity, rapid detection, and versatility compared to the current gold-standard techniques [49].

Biosensors are devices that can measure biological or chemical reactions through the generation of signals that are proportional to the concentration of the analyte tested. Biosensors have wide-scale commercial applications, which include food safety, metabolite detection, disease detection, etc. [50]. The most common types of biosensors are electrochemical, optical, thermal, piezoelectric, and potentiometric biosensors [51,52]. Typically, a biosensor consists of a recognition element and a transducer. Recognition elements are biomolecules that have specific binding properties related to the analyte. Enzymes, antibodies, deoxyribonucleic acids (DNA), and molecularly imprinted polymers (MIPs) are all examples of commonly used biorecognition elements [53]. The generation of a signal from the interaction between an analyte and the bio-receptor is coined as biorecognition [54]. A transducer can convert a biorecognition event into a measurable signal, which may be used to deduce the presence of an analyte and its concentration [55]. Another similar category of sensing technologies is covered by biomimetic sensors, which, similarly to biosensors, generate a signal after an interaction with an analyte. In addition, biomimetic sensors’ working principle is based on the introduction of biomaterials designed to imitate the function of biological processes, i.e., lipid membranes, polymers, etc. [56].

Optical biosensors are the most common type of biosensor used commercially. Detection of analytes by optical biosensors is based on the interaction of an optical field with a biorecognition element [57]. Generally, optical biosensors may be divided into two categories: label-based and label-free [58]. Label-based sensing relies on the use of a label and thus the optical signal is usually generated by a colorimetric, fluorescent, or luminescent response [59]. An example of this type of sensing is the detection of glucose through enzymatic oxidation, which is applied in the most common commercially used biosensor in the world, the hand-held glucose meter used by diabetics to test blood sugar levels [60,61]. In contrast, label-free biosensors generate an electrical signal directly through the interaction of the analyte with the transducer [62]. An example of this is surface plasmon resonance-based optical biosensors (SPR biosensors), which are among the most common biosensors for the detection of pathogens in food and water [63].

Despite the consistent growth of optical biosensors as a solution for antibiotic detection, they still present numerous issues. One of the key challenges that optical biosensors face is cost and reusability [64]. The commercialization and miniaturization of optical biosensors are very limited due to their optical components being extremely delicate and expensive [65]. In this scenario, electrochemical biosensors were shown to be a solid alternative for the detection of antibiotics in a wide variety of samples and matrices, paving the way for the possibility of creating versatile devices that can be employed in resource-limited environments or, more generally, applied for point-of-care analysis thanks to their portability [66]. While a few review articles have already been published on electrochemical biosensors for antibiotic detection, this work focuses on novel biorecognition elements used for the manufacture of electrochemical biosensors and offers an overview of the technologies used for antibiotic detection for commercial purposes, thereby providing fresh insights and expanding the understanding of their potential in diverse and emerging contexts [67,68,69]. The research papers and reviews that are included in this manuscript were mainly published from 2020 to 2024, presenting an updated overview of the most recent works related to electrochemical biosensors.

## 2. General Principles of Electrochemical Biosensors

**Figure 1:**
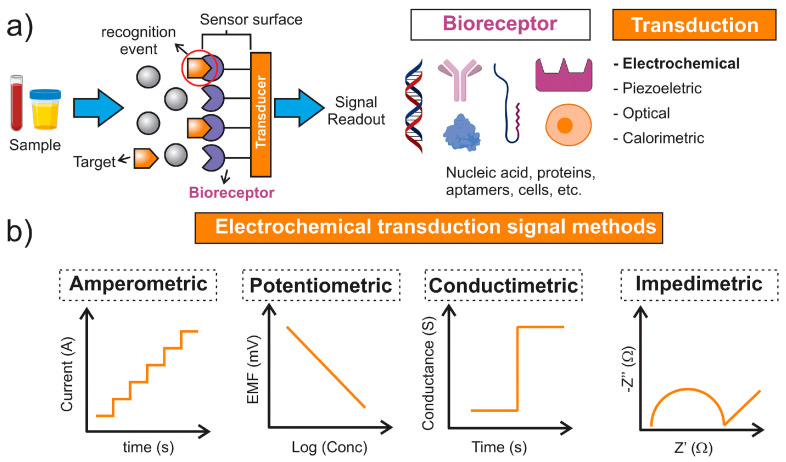
Schematic representation of (**a**) a generic biosensor device showing a sample containing the target interacting with the bioreceptor. After the recognition, the transducer is responsible for generating a signal. (**b**) examples of electrochemical transduction signals. Copyright Sensors and Actuators Reports. 2022 [71].

Electrochemical biosensors are analytical devices used in various fields, including food safety, environmental monitoring, and medical and industrial diagnostics. These sensors are mainly composed of four elements: (a) a recognition element, whose function is to selectively interact with the target molecule, (b) a transducer, responsible for the conversion of the signal generated after the interaction of the target with the recognition element into a measurable output, (c) an electrolyte, namely the medium in which the electrochemical reaction takes place, and (d) the electrodes, which are divided into reference, counter, and working electrodes and can be made of different materials including carbon, gold, or other conductive materials. To date, there are multiple types of electrochemical biosensors, including amperometric, potentiometric, and impedimetric biosensors. Electrochemical-based signals generate either a measurable current (amperometry), a measurable potential or charge accumulation (potentiometry), a measurable change in impedance (electrochemical impedance spectroscopy, EIS), or measurably alter the conductive properties of a medium (conductometry) between electrodes [70]. A schematic representation of the working principle of an electrochemical biosensor is provided in Figure 1 [71]. Therefore, the aim of this chapter is to elucidate the working principles of the previously mentioned electrochemical analysis techniques.

### 2.1. Amperometric Biosensors

Amperometry is an electroanalytical technique that involves the application of a constant reducing or oxidizing potential to an indicator working electrode and the subsequent measurement of the generated current. Typically, the magnitude of the measured current is dependent on the concentration of the reduced/oxidized species. In the case of biosensors, the reduction or oxidation of an electroactive species is directly proportional to the concentration of the analyte being investigated [72]. In this scenario, amperometric biosensors are a type of electrochemical biosensor that enables the quantification of an analyte within a sample matrix by transducing surface-level interactions caused by an electroactive species into a current signal [73]. The intrinsic simplicity of the transducer makes these biosensors applicable for low-cost portable devices [74]. An amperometric transducer is used to study the charge transfer at the interfaces of phases, typically between two electrodes separated by an electrolyte. These types of systems are called electrochemical cells and are used to describe the system in which the reaction takes place. One of the half-cells of the electrochemical compartment is heavily controlled to study the charge transfer occurring at the other half-cell, known as the working electrode. Different amperometric methods can be used in electrochemical biosensors, including cyclic voltammetry (CV), differential pulse voltammetry (DPV), or square wave voltammetry (SWV), with the final two being used in most commercial products (such as glucose tests) [73].

### 2.2. Potentiometric Biosensors

Potentiometry is an electrochemical technique similar to amperometry; however, to conduct potentiometric analysis, the potential difference between a reference electrode and an indicator electrode inside of an electrolyte is measured to extract information about bioreceptor–analyte interactions [75]. This technique has several advantages compared to amperometry as it requires little power and can be compact and portable. In potentiometric biosensors, at zero current, the potential changes are correlated to the changes in concentration of a certain analyte. The electromotive force (EMF), namely a measure of the energy provided by a power source per unit charge and better known as the voltage of a cell, is dependent on the concentration. This relationship may be related using the Nernst Equation (Equation (1)) [76].

(1) $$E_{cell}=E_{cell}^{{}^{\circ}}-\frac{RT}{zF}\ln Q$$

In this equation, E_cell_ represents the standard electrode potential of the cell, measured in Volt, R is the universal gas constant, T is the temperature in Kelvin, z is the ion charge (moles of electrons), F is Faraday’s constant, and Q is the reaction quotient.

### 2.3. Coulometric Biosensors

The coulometric analysis uses either an applied current or a potential to exhaustively convert an analyte from one oxidation state to another at the working electrode. This method measures the total current passed, either directly or indirectly, and uses this to determine the number of electrons exchanged through the electrochemical cell. As a result, Faraday’s law may be used to extract the concentration of the analyte based on the number of electrons passed (Equation (2)) [77].

(2) $$Q=nFN_{a}$$

where n is the number of electrons per mole of the analyte, F is Faraday’s constant, and N_a_ represents the moles of the analyte. This relationship is valid when 100% of the applied current is used to change the oxidation state of the analyte, which is typically the case as the analyte’s conversion rate when passing through the electrodes is nearly 100%, so slight losses of current are negligible [78]. An advantage of coulometric analysis relative to other techniques is that it lacks the need for calibration curves or chemical standards for the quantification of the analyte, making it an absolute method [78].

### 2.4. Impedimetric Biosensors

Electrochemical impedance spectroscopy (EIS) is an analytical technique where the impedance or resistance of a system after the target–recognition site interaction is measured in Ohm [79]. EIS offers numerous advantages compared to other electrochemical sensing techniques, as it can utilize small signal analysis by investigating sinusoidal signal relaxations over a wide range of frequencies, from approximately 0.1 Hz to 1 MHz [76]. EIS may be used to analyze charge transfer, mass transfer, and diffusion processes. Furthermore, EIS has the capability of examining intrinsic material properties or processes taking place that will impact the conductance, resistance, or capacitance of a cell [80]. Analyzing impedance differs from resistance, as in a cell with a direct current (DC) applied, resistance obeys Ohm’s law whereas impedance does not. This is due to resistance opposing the flow of both DC and alternating current (AC), whereas impedance solely opposes the flow of AC [81]. By varying the excitation frequency of the applied potential over a range of frequencies, it is possible to calculate the complex impedance, which is the sum of the real impedance (resistance, which measures the opposition to a flowing current) and imaginary impedance (reactance, which measures the opposition to a change in current) of the system as a function of the frequency [70]. Consequently, the analysis of the impedimetric signals generated after the detection at the interface of a selected target can provide information about the presence of a molecule in a sample and lead to quantitative data on the target molecule itself [82]. 

## 3. Types of Electrochemical Biosensors Used for Antibiotic Detection

The method of transduction used in an electrochemical biosensor is very dependent on the type of recognition element [83]. The most common biorecognition molecules used are MIPs, antibodies, nucleic acids, aptamers, and enzymes, which are used due to their specific binding capabilities and activity [70]. The aim of this chapter is to elucidate the key biorecognition molecules used in electrochemical biosensors for antibiotic detection, highlighting the positive aspects of each sensing type, such as LoDs and durability. An overview of the biorecognition elements with their main advantages and disadvantages is provided in Table 1 [53].

### 3.1. Aptamer-Based Electrochemical Sensors

Aptamers are specialized molecules, typically short strands of single-stranded DNA or RNA. Due to their unique three-dimensional structures, aptamers can bind to a wide variety of targets, including ions, small molecules, proteins, and even cells, with high specificity and selectivity [84]. Aptamers are synthesized in vitro through a systematic process known as SELEX (Systematic Evolution of Ligands by EXponential enrichment) [85]. This process involves the repetitive binding, separation, and amplification of oligonucleotides to selectively produce aptamers with a high affinity for specific target molecules. The SELEX process allows for the large-scale production of uniform and high-fidelity aptamers [86]. The synthetic nature of aptamers confers several advantages, including high stability, ease of modification, and the ability to withstand harsh conditions, making them ideal for use in biosensors [87].

**Figure 2:**
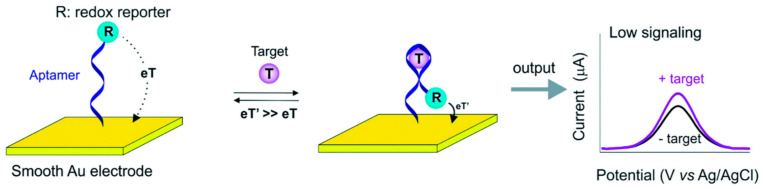
Schematic illustration of the working principle of electrochemically based aptamer sensors. Copyright Toxics 2023 [91].

An example of aptamer implementation in biosensor technology, particularly in electrochemical sensors, involves the formation of covalent bonds between the aptamer and the sensor’s surface [88]. This covalent attachment ensures a stable and oriented immobilization of the aptamers on the electrode surface, enhancing the sensor’s specificity and sensitivity [89]. The binding event between the aptamer and its target leads to a detectable change in the electrochemical signal, which can be quantitatively measured (Figure 2) [90].

**Figure 3:**
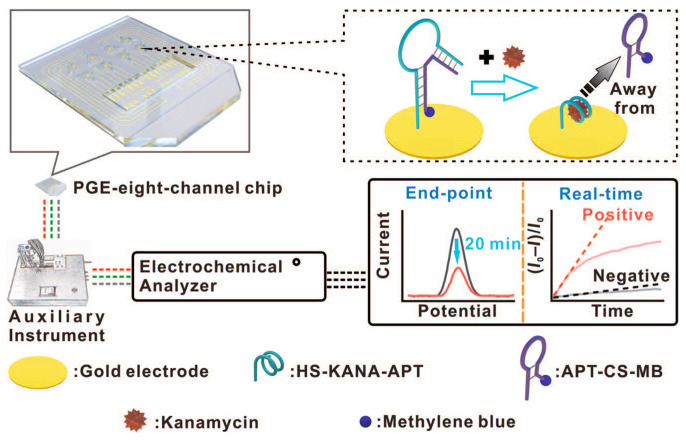
Portable aptamer-based sensor for kanamycin developed by Bao et al. Copyright Chinese Jour. Of Chem., 2023 [93].

This method capitalizes on the specific interaction between the aptamer and the antibiotic to facilitate accurate and sensitive detection, illustrating the practical application of aptamers in the development of advanced biosensing technologies [92]. An interesting recent example of the utility of aptamers in electrochemical biosensors was published by Bao et. al. They developed a sensor for kanamycin on the surface of an integrated portable plastic gold electrode, as shown in Figure 3, resulting in a sensor with good stability and a low LoD of 0.40 μmol/L, which is highly effective for the detection of kanamycin in environmental water samples [93].

**Figure 4:**
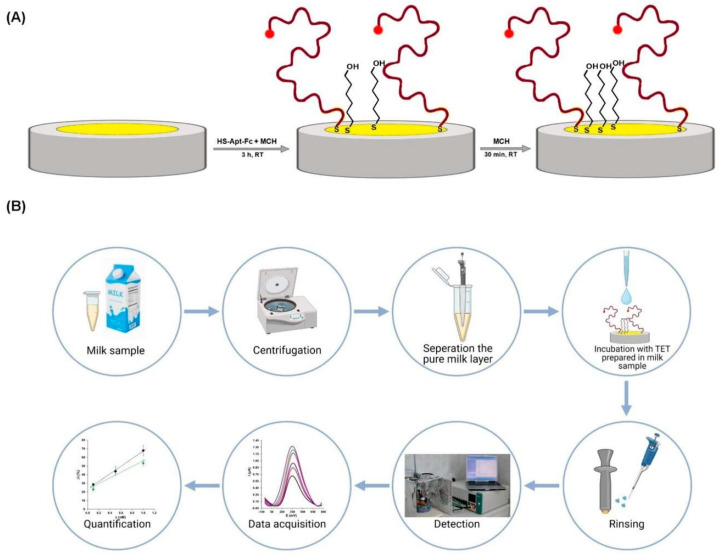
A modified ssDNA aptamer sensor for the selective detection of tetracycline designed by Malecka-Baturo et al. Copyright Int. J. Mol. Sci. 2022 [94]. (**A**) Scheme of the aptasensor preparation, and (**B**) representation of the steps needed to use the aptasensor.

The work published by Malecka-Baturo et. al. is another example illustrating that low LoDs can be achieved with aptamer-based sensors. They utilized a 5′-ferrocene-modified ssDNA aptamer to develop a sensor for tetracycline detection in cow milk samples, as depicted in Figure 4. They achieved a LoD of 0.20 nmol/L with high selectivity for tetracycline over structurally related derivatives [94]. 

More examples of recently developed technologies based on aptamer biosensors, with relative target molecules, detection methods used, LoDs, and type of sample analyzed, are summarized in Table 2.

### 3.2. Molecularly Imprinted Polymer-Based Electrochemical Sensors

**Figure 5:**
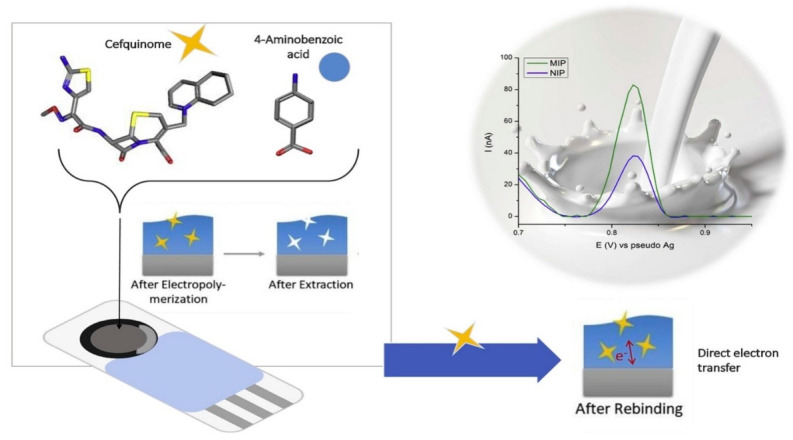
Schematic representation of the extraction and rebinding process on an antibiotic (cefquinome) after electropolymerization on the surface of an electrode and consequent electrochemical analysis. Copyright Sensors and Act. B: Chemical, 2019 [108].

Molecularly imprinted polymers (MIPs) represent a class of highly specific polymeric materials crafted for recognizing and binding selectively to target molecules. The unique nature of MIPs lies in their synthesis process, during which a target molecule, known as the template, is mixed with functional monomers. These monomers form reversible bonds with the template due to the presence of weak forces such as hydrogen bonding, ionic interactions, π–π interactions, and acid–base interactions [106]. This arrangement is stabilized by a cross-linking agent, leading to the formation of a three-dimensional polymer matrix, typically initiated by a free radical polymerization mechanism. Numerous polymerization techniques can be used such as bulk, precipitation, emulsion, and suspension polymerization, which alter specific elements of the synthesized MIP such as particle size, sensitivity, selectivity, etc. [107]. Post-polymerization, the template is carefully extracted, leaving behind a polymeric structure with cavities containing a specific size, functional groups, and shape to fit a specific template molecule (Figure 5).

The most common application of MIPs is their combination with a portable transducer element. An example is screen-printed electrodes (SPEs), where MIPs synthesized a priori are integrated into the sensor’s surface for specific analyte detection. Nonetheless, it is becoming more and more common to implement the molecularly imprinted polymer technology directly onto the surface of interest, namely on the surface of an electrode or another material that can be used to perform electrochemical measurements. These are known as surface imprinted polymers (SIPs), which are a subclass of MIPs designed to have selective recognition sites on their surface. A key feature of SIPs is that a template molecule is imprinted directly on or near the surface of a polymer matrix, which enhances the accessibility and binding efficiency of the imprinted sites to a specific analyte. Consequently, a higher surface area-to-volume ratio is achieved, leading to improved sensitivity and binding kinetics. To this end, the functionalization is carried out via electropolymerization on the electrode’s surface, resulting in a faster and more reproducible preparation of the sensor itself [109]. Moreover, the direct functionalization of the surface reduces the batch-to-batch variations that can be present in higher amounts if an extra step (i.e., polymer synthesis) must be carried out in advance. It is difficult to categorize SIP-based electrochemical biosensors as a class of their own since they are mainly used for the detection of bigger molecules or full cells. Therefore, they have been implemented in Table 3 and combined with an overview of the recent advances for MIP-based electrochemical biosensors for antibiotic detection.

**Figure 6:**
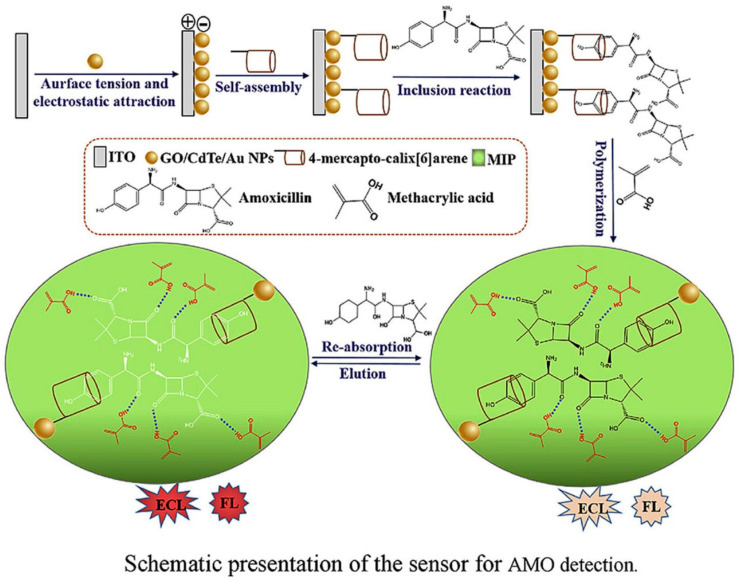
The dual recognition sensor for amoxicillin. Copyright Analytica Chimica Acta, 2020 [110].

A representative example MIP-based sensor is the dual recognition sensor for the detection of amoxicillin shown in Figure 6. Li et al. combined electrochemiluminescence with fluorescence to obtain a highly sensitive sensor with a LoD of 8.3 × 10^−12^ mol/L [110].

**Figure 7:**
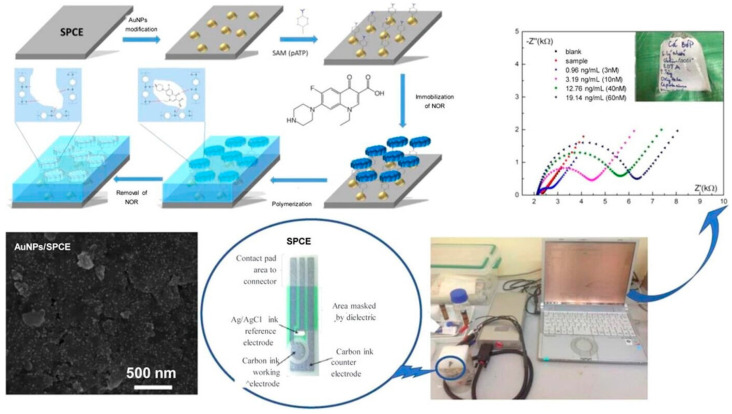
Overview of the norfloxacin sensor developed by Thi Vu et al. [111]. Copyright ACS Omega, 2023.

A second interesting MIP-based sensor was developed by Thi Vu et al. They were able to make a selective sensor for the detection of norfloxacin in pharmaceutical and aquacultural samples. Through the incorporation of Au nanoparticles into the MIP, as shown in Figure 7, they achieved a LoD of 0.15 ng/mL [111].

**Table 3:** Molecularly imprinted polymer-based electrochemical biosensors for antibiotic detection.

Sensor Type	Target Molecule	Method of Detection	Limit of Detection	Sample	Monomer	Ref.
SPE combined with an MIP prepared from dual functional monomers.	Erythromycin Clarithromycin Azithromycin	DPV	1.1–1.6 nM	Buffer, Tap water samples	m-phenylenediamine	[112]
Gold screen-printed electrode (Au-SPE) functionalized via electropolymerization of custom-made conjugated monomer (Th_2_-NDI-PIA)	Streptomycin sulfate	DPV	0.190 pM	Buffer, tap water	Th_2_-NDI-PIA	[113]
Electropolymerized MIPs onto a screen-printed carbon electrode (SPCE)	Azithromycin	DPV	0.08 µM	Water samples	4-aminobenzoic acid	[114]
MIP electrodeposited onto the surface of AuNPs/rGo/single-walled carbon nanotube-modified GCE	Pefloxacin	DPV	16 nM	Milk	o-phenylenediamine (oPD)	[115]
CO_2_ laser-induced graphene (LIG) with AuNPs and MIPs	Tetracycline	DPV	0.32 nM 0.85 nM 0.80 nM	Buffer, Milk, Meat	oPD	[116]
Nanocomposite molecularly imprinted polymer (nanoMIP) using oxidised MWNCTs and ultrathin overoxidised polypyrrole MIP	Sulfamethoxazole	DPV	0.41 μM	Buffer	pyrrole	[117]
Dual recognition MIP-coated graphene oxide loaded with CdTe quantum dots/AuNPs (GO/CdTe/AuNPs) on an indium tin oxide (ITO) electrode	Amoxicillin	DPV EIS	8.3 pM	Buffer	α-methacrylic acid	[110]
Gold nanoparticles (AuNPs) and MIP-based electrochemical sensors	Norfloxacin	EIS	0.15 ng/mL	Aquaculture water	4-aminothiophenol	[111]
Bifunctional dual-template molecularly imprinted polymer-modified electrode	Ceftazidime Avibactam	EIS SWV	35 μM 0.5 μM	Serum samples	o-PD	[118]
Sensor using magnetic nanoparticles (mag) and molecularly imprinted polymer	Tetracycline	SWV	0.15 μM	Milk	Acrylic acid	[119]
Molecularly imprinted electrochemiluminescence (ECL) sensor using amino-functional titanium carbide nanodots (TNDs) and carbon nitride nanosheets (CNNS)	Ciprofloxacin	ECL EIS	1.2 nM	Food samples including chicken, milk, and pork	o-PD	[120]
MIP sensor using electro-polymerization with surface-deposited AuNPs	Sufhaguanidine Sulfamerazine	CV DPV CA	0.030 µM	Human fluids	oPD	[121]
Chitosan gold nanoparticle-decorated MIP (Ch-AuMIP) modified GCE	Ciprofloxacin	CV	0.210 µM	Water	Methacrylic acid (MAA)	[122]
MIP electrochemical sensor using Fe_3_N-Co_2_N nanoarray with high electric conductivity and large surface area for MIP growth	Ampicillin	CV EIS	0.365 nM	Milk samples	N-N-dimethyl bisacrylamide	[123]
MIP coated on graphene oxide deposited as a thin film on GCE	Amoxicillin	CV DPV	0.294 nM	Buffer	APTES + PTES	[124]
Fe-doped porous carbon (Fe-PC)-modified Au electrode covered with MIP film electropolymerized onto an electrode	Lomefloxacin	CV DPV EIS	0.2 nM	Water samples	o-PD	[125]
MIP-based biomimetic layer electrodeposited onto a glassy carbon electrode (GCE)	Azithromycin	CV EIS	0.85 nM	Spiked plasma, tears, and urine samples	3-thienyl boronic acid	[126]
Aggregation-based ECL sensor using ferriferous oxide@Pt NPs for signal amplification	Ciprofloxacin	CV EIS	0.60 pM	Meat samples	4-aminothiophenol	[127]
Screen-printed electrode	Erythromycin	CV EIS	0.1 nM	Buffer	MAA	[128]

### 3.3. Antibody-Based Electrochemical Sensors

Antibody-based electrochemical biosensors, also known as electrochemical immunosensors, leverage the specific affinity of antibodies for antigens to detect and quantify target analytes in a sample [129]. The core functionality of these biosensors involves immobilizing antibodies onto an electrode surface, acting as biorecognition elements [130]. Similarly to MIP sensors, SPEs are an attractive transducer option for immunosensors due to their ability to be mass-produced, their low cost, and their applications in portable detection devices [131]. The binding of these antibodies to their corresponding antigens induces an electrochemical signal, which is measured and correlated with the antigen’s concentration in a sample. Often, labeling components such as nanomaterials are typically employed for the detection of an analyte using immunosensors [132,133]. A common technique is the use of a redox mediator which is catalytically oxidized in the presence of an enzyme substrate [134].

**Figure 8:**
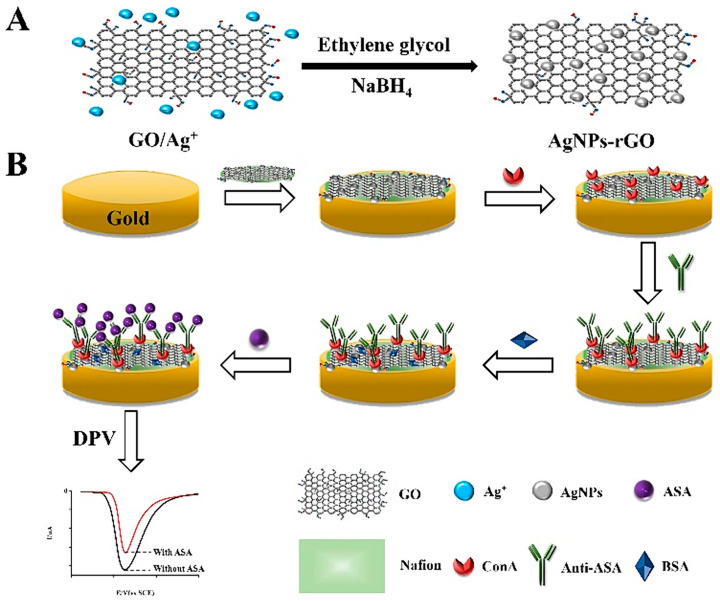
Working principle of the ConA arsanilic acid developed by You et al. Copyright J. Electroanalytical Chem., 2024 [135].

A recent example is the use of concanavalin A (ConA), which showed excellent binding ability with monoclonal antibodies of arsanilic acid through lectin–sugar interactions. The working principle of the sensor is shown in Figure 8. You et al. developed an Ag nanoparticle-reduced graphene oxide nanocomposite which showed a LoD of 0.011 ng/mL [135].

There are numerous types of antibodies used for electrochemical immunosensor applications, including monoclonal, polyclonal, recombinant antibodies, etc. Some examples of their application for the detection of antibiotic residues are reported in Table 4.

## 4. Commercially Available Sensors for Antibiotic Detection 

Detecting antibiotic residues is essential for maintaining food safety, monitoring the environment, and performing clinical diagnostics. Therefore, the main focus of this chapter is to present the technologies that are commercially used to detect the presence of antibiotics in different types of samples, flowing out to a discussion on electrochemical biosensors and electrochemical devices that have been recently introduced on the market. To date, various commercially available sensors have been developed to address this need, offering different mechanisms of detection and varying degrees of sensitivity, specificity, and practicality. In this chapter, we discuss four main categories of these technologies for the detection of antibiotics, namely (a) gold-standard technologies, (b) optical-based sensors, (c) immunoassay-based sensors, and (d) emerging electrochemical-based biosensors and devices.

### 4.1. Gold-Standard Technologies

A gold standard technology is a technique that has been thoroughly tested and has proven to be highly reliable. Because of the need to use effective methods for the detection of antibiotics in various samples, traditional methods such as high-performance liquid chromatography (HPLC), mass spectrometry (MS), microbiological assays, capillary electrophoresis (CE), and ultraviolet–visible (UV–Vis) spectroscopy are highly regarded and used thanks to their accuracy, sensitivity, and reliability. High-performance liquid chromatography (HPLC) combined with mass spectrometry (MS) is known for its precision in antibiotic detection. Companies such as Agilent Technologies, Santa Clara, CA, USA and Thermo Fisher Scientific, Waltham, MA, USA produce advanced HPLC-MS systems. For example, Agilent’s 1290 Infinity II LC system offers excellent resolution and throughput, while Thermo Fisher’s TSQ Quantis MS provides accurate quantification of antibiotics at very low levels (parts per billion) [146,147]. On the other hand, microbiological assays are another important technology, valued for their ability to detect a wide range of antibiotics through microbial growth inhibition. Neogen Corporation, Lansing, MI, USA and Charm Sciences, Lawrence, MA, USA are key providers in this area. Neogen’s Soleris Next Generation (NG) system and Charm Sciences’ ROSA (Rapid One Step Assay) platforms are extensively used in the food and dairy industries, delivering results within hours and detecting antibiotic residues at levels of low parts per billion [148,149]. Furthermore, capillary electrophoresis (CE) is another reliable method for analyzing antibiotics. In this scenario, the company Sciex, Framingham, MA, USA introduced the PA 800 Plus Pharmaceutical Analysis System, which achieves high-resolution separation and quantification of antibiotics in various samples, with detection limits in the low nanogram per milliliter (ng mL^−1^) range [150]. Ultraviolet–visible (UV–Vis) spectroscopy is another conventional technique used for antibiotic detection. As an example, Shimadzu’s UV-1900i UV–Vis Spectrophotometer (Kyoto, Japan) is widely used due to its accuracy in detecting various antibiotic compounds, with detection limits typically in the microgram per liter (μg L^−1^) range, which is slightly higher compared to other devices, although it offers a high degree of reliability [151]. In the last decades, many companies have introduced important technologies and products to the market with the aim of detecting the presence of antibiotics in a variety of samples. Some examples with their related working parameters are summarized in Table 5.

These traditional methods, supported by products from leading companies, provide highly sensitive, specific, and reproducible results, making them essential tools for monitoring and detecting antibiotic residues across a range of samples. Nonetheless, despite their impressive capabilities, the cost and portability of these technologies can pose significant challenges, particularly in resource-limited settings where access to sophisticated equipment and funding is constrained. This financial barrier and the often bulky nature of these devices limit their widespread application and practicality in such areas. As a result, researchers and engineers are actively exploring novel materials, design methodologies, and fabrication techniques to create next-generation technologies that are not only cost-effective but also highly portable. This ensures that these critical diagnostic tools can be installed more broadly, meeting the diverse needs of various applications and geographic regions, thus bridging the gap in accessibility and effectiveness.

### 4.2. Optical-Based Sensors

Optical-based sensors utilize the interaction of light with a sample to detect the presence of antibiotics. These sensors often are based on principles such as fluorescence, absorbance, and surface plasmon resonance (SPR). Commercially available optical sensors are valued for their high sensitivity and specificity. In fact, the market for optical-based sensors has grown significantly, providing rapid and sensitive detection methods. Several companies lead this field with innovative products, each offering unique performance metrics and catering to specific industry needs. As an example, Charm Sciences Inc. stands out with its Charm II System, which is particularly effective in the dairy industry [158]. This system can detect a wide range of antibiotic residues in milk and dairy products within eight minutes. With a limit of detection (LoD) of 1ppb for several antibiotics, the Charm II System combines speed and sensitivity, making it a valuable tool in ensuring food safety. Furthermore, the company R-Biopharm AG, Pfungstadt, Germany, offers the RIDAQUICK SULFONAMIDES test kit, which provides qualitative analysis of sulphonamide residues in meat and other food matrices [159]. The test delivers results in ten minutes with a LoD of 10 ppb, making it a quick and reliable choice for food safety testing. Another example of commercially available products based on optical biosensors is given by the company Neogen Corporation, where the product BetaStar Advanced is designed primarily for the dairy industry, offering results in just five minutes [160]. This product is selective for the detection of beta-lactam antibiotics with a LoD of 2 ppb. Thermo Fisher Scientific’s QExactive Orbitrap, although primarily a mass spectrometer, includes optical detection capabilities [161]. This versatile instrument can detect antibiotic residues in food and environmental samples with a LoD ranging from low ppb to ppt [162]. Besides being an expensive alternative, the results are available in less than an hour, making it suitable for high-throughput testing. Similarly, IDEXX Laboratories’ SNAP Tetracycline Test (Westbrook, ME, USA) targets milk testing, detecting tetracycline residues within ten minutes with a LoD of 4 ppb [163].

Overall, the market for optical-based sensors for detecting antibiotic residues is diverse, with leading companies offering products that vary in performance metrics, including detection limits, time to results, and pricing. These sensors provide essential tools for ensuring food and environmental safety, supporting the specific needs of various industries.

### 4.3. Immunoassay-Based Sensors

Immunoassay-based sensors are highly valued for their exceptional specificity and sensitivity in detecting antibiotic residues in a variety of sample types, including food, water, and biological tissues. These sensors utilize the precise binding interaction between antigens and antibodies to deliver quick and dependable results. By exploiting this high specificity, immunoassay-based sensors can accurately identify and quantify antibiotic residues in complex samples, ensuring both rapid detection and reliable performance. Several companies have developed innovative immunoassay-based products, each tailored to meet the specific needs of different industries. Therefore, this paragraph explores key players in this field and their notable products, discussing performance metrics such as time required for measurement, limit of detection (LoD), and pricing. R-Biopharm AG is a prominent competitor in the immunoassay market, offering products such as the RIDASCREEN Chloramphenicol [164]. This ELISA-based sensor is designed to detect chloramphenicol residues in various food products, providing results within two hours. With a limit of detection of 0.05 ppb, the RIDASCREEN Chloramphenicol offers high sensitivity, ensuring the reliable detection of this antibiotic. Furthermore, the company Perkin Elmer, Waltham, MA, USA offers the MaxSignal Beta-lactam ELISA Test Kit, designed for detecting beta-lactam antibiotics in food samples [165]. This immunoassay-based sensor provides results within two hours and presents a LoD of 1 ppb. Another example is given by the company Enzo Life Sciences, Farmingdale, NY, USA, which provides the Amp’d ELISA Kit for the detection of aminoglycoside residues in food and animal feed [166]. This immunoassay-based sensor offers results in under two hours with a LoD of 0.1 ppb, ensuring high sensitivity and specificity, making it a valuable tool for ensuring compliance with regulatory standards. Together with the aforementioned examples, other products based on similar technology are also present on the market. Some examples of the most significant ones are reported in Table 6.

In summary, immunoassay-based sensors are essential tools in the detection of antibiotic residues, offering high specificity and sensitivity across various sample types. Some leading companies have developed robust products that satisfy the needs of different industries. These sensors vary in their performance metrics, including detection limits, time to results, and pricing, providing versatile and effective solutions for ensuring public health and safety. Nonetheless, the cost of these technologies and their portability can still be considered a drawback, especially if the application needs to be performed in resource-limited areas. Therefore, both academia and industry are continuously researching novel alternatives to ensure that the technologies can service all needs.

### 4.4. Electrochemical-Based Biosensors

Electrochemical-based biosensors are a prominent category among commercially available sensors for antibiotic detection. These devices are characterized by cost-effectiveness, portability, and good precision, thus offering a robust alternative to gold-standard technologies or conventional detection methods, which, despite their accuracy, can have drawbacks such as high operational costs and complex preparatory requirements. For example, sensors developed by companies such as Randox Food Diagnostics, Crumlin, UK provide rapid and reliable detection of various antibiotics, helping to maintain food quality and safety [175]. Environmental monitoring also benefits from these biosensors, as they are used to detect antibiotic contamination in water sources. Moreover, companies such as Bio-Rad Laboratories, Hercules, Ca, USA, Thermo Fisher Scientific, and Clinical Diagnostics (Bangkok, Thailand) utilize electrochemical-based sensing detectors to monitor antibiotic levels in biological samples, assisting in effective treatment management and preventing antibiotic resistance. However, there are other examples of spin-off companies and products developed for the same purpose (Table 7).

In addition, there are some examples of commercially available products for the specific detection of various types of antibiotics. The devices listed in Table 7 are not only particular tools that can be used on-site for sensing purposes but also more general apparatus that can be applied to the detection of antibiotic residues. In particular, the Dropsens Screen-Printed Electrodes are examples of electrochemical devices that can be functionalized differently based on the needs of the user and then coupled with specific appliances such as a potentiostat to be able to obtain the desired measurements (i.e., cyclic voltammetry, differential pulse voltammetry, etc.). 

Overall, the introduction of electrochemical-based devices is becoming increasingly prevalent in the sensing field thanks to their versatility and ease of use. These devices offer a wide range of applications, from environmental monitoring to medical diagnostics, providing accurate and reliable measurements. Nonetheless, besides being cheaper compared to almost all the gold-standard technologies, some options can still be considered expensive, posing a barrier to widespread adoption. However, developing highly portable technologies, such as the Sensit Smart from PalmSens, Utrecht, The Netherlands, is opening the way toward more accessible and mobile sensing applications. This portability allows for on-site testing and real-time data collection, significantly enhancing the practicality and efficiency of sensing processes across various fields.

## 5. General Conclusion and Future Perspectives

Looking forward, the integration of electrochemical-based devices into the sensing field marks a significant technological advancement, driven by their versatility and user-friendly nature. These devices, renowned for their wide array of applications, have become valid alternatives in domains ranging from environmental monitoring to medical diagnostics. They provide accurate and reliable measurements that are crucial for making informed decisions and ensuring public safety, overcoming some of the main issues related to the gold-standard technologies already present on the market. In fact, the key advantage of electrochemical sensors lies in their ability to deliver precise data quickly and efficiently. For instance, in environmental monitoring, these devices can detect pollutants at very low concentrations, enabling timely interventions to prevent ecological damage. In medical diagnostics, electrochemical sensors are not only used for the detection of antibiotics but also to monitor glucose levels, detect biomarkers for various diseases, and even assess the efficacy of treatments in real-time. This capability is particularly beneficial for patients who require constant monitoring, as it allows for better management of their conditions and reduces the need for frequent hospital visits. When comparing different kinds of electrochemical biosensors, it is important to consider their sensitivity, cost, and practical applications. For example, amperometric sensors are known for their high sensitivity and fast response times, making them ideal for real-time monitoring. However, they may be more expensive to produce due to the need for specialized electrodes. Potentiometric sensors, on the other hand, are generally more cost-effective but might offer lower sensitivity in comparison. Conductometric sensors, which measure changes in electrical conductivity, provide a balance between cost and sensitivity but may require more complex calibration procedures. Despite their many benefits, the adoption and marketing of electrochemical-based devices is not without challenges. While these sensors are generally more affordable than many traditional gold-standard technologies, the initial cost can still be prohibitive for some users. Furthermore, the ongoing advancements in sensor technology are likely to drive down costs and improve accessibility even further. Innovations in materials science, such as the use of more affordable and sustainable materials for sensor construction, are expected to make these devices even more economical. Additionally, improvements in manufacturing processes can enhance the scalability of sensor production, leading to lower prices and wider availability.

The high cost can limit the widespread adoption of these sensors, particularly in low-resource settings where budget constraints are a significant concern. Therefore, while the cost-effectiveness of electrochemical sensors is an advantage, further efforts are needed to reduce costs and make these technologies more reliable and accessible to a broader audience.

## Figures and Tables

**Table 1:** Advantages and disadvantages of specific biorecognition elements for biosensors [53].

Biorecognition Element	Advantages	Disadvantages
Antibody Enzyme	Selectivity, Reusability, High specificity, Versatility	Reproducibility, Batch variations, Processing stability, Cost; Limited shelf-life
Nucleic Acids Aptamers	Sensitivity, Reproducibility	Cost, Non-specific binding interactions, Stability
Molecularly imprinted polymers	Stability, Reusability, Low cost	Reproducibility, Template removal from cavities
Surface imprinted polymers	Selectivity, Robustness	Scalability, Template availability

**Table 2:** Electrochemical aptasensors for antibiotic detection.

Sensor Type	Target Molecule	Method of Detection	Limit of Detection	Sample	Ref.
Modified nanocomposite including multi-walled carbon nanotubes (MWCNTs), gold nanoparticles (AuNPs), reduced graphene oxide (rGO), chitosan (CS), and modified nanosheets to bind with OTC-specific aptamer	Oxytetracycline	DPV	30 pM	Spiked milk samples	[95]
Dual-labeled multiple aptasensor using RNA-based aptamer strands, semiconductor quantum dots (QDs), and gold nanoshells (AuNSs). Conjugation of biotinylated aptamers to SA-coated sadmium sulfide (CdS) and lead sulfide (PbS) QDs.	Kanamycin Tobramycin	DPV	0.12 nM 0.49 nM	Spiked milk	[96]
Aptamer (Apt) BSA/Apt/indole/MWCNTs/GCE with the synergistic amplification of multi-walled carbon nanotubes (MWCNTs) and indole	Sulfadoxine	DPV	0.033 μM	buffer	[97]
Novel 3D honeycombed goldnanovoids@aptamer nanostructured platform with ferrocene labeling on aptamer strands	Tetracycline	DPV	1.2 nM	Wastewater	[98]
Aptamer sequence bonded onto bismuth oxide nanofibers paired with AuNPs	Ampicillin	DPV	0.88 nM	Water and milk	[99]
Exonuclease III (Exo III)-aided target-aptamer binding recycling (ETBR) activated bipedal DNA machine	Kanamycin	CV DPV	7.1fM	buffer	[100]
Polyethyleneimine grafted rGO and titanium dioxide nanocomposite material	Ciprofloxacin	DPV EIS	0.7 nM	Real water samples	[101]
Aptasensor prepared using AuNPs combined with ferroferric oxide-multi walled carbon nanotube (Fe_3_O_4_-MWCNTs) complex	Penicillin antibiotics (PENs)	CV DPV EIS	0.667 nM	Spiked milk samples	[102]
DNA aptamer and partially complementary short chain assembled onto integrated portable plastic gold electrode (PGE) through Au-S bonds.	Kanamycin	SWV CV	0.40 μM	Buffer	[93]
Thiolated aptamer labeled with ferrocene covalently co-immobilized onto a gold electrode surface with 6-mercaptohexan-1-ol	Tetracycline	SWV	0.20 nM	Spiked milk	[94]
Nanocomposite comprising a functionalized MOF, a MWCNT@reduced graphene oxide nanoribbon, and a covalent organic framework (COF). cDNA strands with terminal amino groups anchored on the surface, as well as penetration into the pores	Kanamycin	SWV CV	13 nM	Fish Meat Milk	[103]
Amplified aptasensor using Immobilized DNA strands on AuNPs/Fe-based metal organic framework (MOF)	Tobramycin	SWV	56 pM	Spiked milk samples	[104]
MOF of Ni^2+^-2,3,6,7,10,11-hexahydroxytriphenylene (Ni-HHTP) coated on a SPE, followed by non-covalent adsorption of tetracycline aptamer (TC-Apt) through the π-stacking	Tetracycline	CV SWV	1.9 pM	buffer	[105]

**Table 4:** Antibody-based electrochemical biosensors for antibiotic detection.

Sensor Type	Target Molecule	Method of Detection	Limit of Detection	Sample	Ref.
A composite o0066 chitosan (CH) and thioglycolic acid capped vanadium sulfide quantum dots (TGA-VS_2_QDs) was constructed on glass substrate coated with ITO film to form electrodes on which monoclonal antibodies (mAb) for amoxicillin were immobilized	Amoxicillin	DPV	1.65 pM	Spiked fish	[136]
Immunosensor based on AgNPs-reduced graphene oxide (AgNPs-rGO) and staphylococcal protein A (SPA) that was targeted to immobilize mAb	Virginiamycin M1	DPV	0.18 ng mL^−1^	Meat	[58]
A cephalexin–bovine serum albumin (CFX-BSA) conjugate was developed to create antibodies (Abs). Graphene quantum dots (GQDs) were used for signal enhancement	Cephalexin	DPV	0.53 fM	Spiked animal source food	[137]
Anti-quinolone Ab immobilized onto screen-printed dual carbon electrodes	Enrofloxacin	DPV	3 ng mL^−1^	Meat	[131]
molybdenum disulfide nanoparticles (nMoS_2_NPs) deposited on ITO-coated glass substrate with Abs bonded through amide linkages	Ampicillin	DPV	0.028 µg mL^−1^	Milk, orange juice, and tap water	[138]
Sensor based on AgNPs-rGO nanocomposite and concanavalin A (ConA) that was bound to mAbs through lectin–sugar interactions	Arsanilic acid	DPV	0.008 ng mL^−1^	Buffer, chicken, eggs	[135]
Nanocomposite-modified glass carbon electrode (GCE) with a biospecific CeO_2_-chitosan (CHIT)-modified nanocomposite on which polyclonal Abs were immobilized	Sulfamethoxazole	DPV	1.3 nM	Buffer or food	[139]
Immunosensor platform based on Ab-conjugated magnetic particles on an electrode surface that uses a 3D cell to accumulate the analyte	Amoxicillin	SWV	0.44 µM	Raw milk	[140]
Origami paper-based analytical device (oPAD) with multiple Ab zones for simultaneous quinolone residue detection	Norfloxacin, Enrofloxacin	SWV	2.02 ng mL^−1^ 1.70 ng mL^−1^	Food	[141]
A graphite composite electrode (GEC), biofunctionalized magnetic μ-particles, and electrochemical nanoprobes prepared by labeling specific antibodies with CdS nanoparticles (*CdS*NP).	Sulfapyridine	SWV	0.11 μg kg^−1^	Honey	[142]
rGO-gadolinium oxide nanocomposite (rGO@Gd_2_O_3_ NC) with suspended mAbs on a SPE	Gentamicin	CV	0.424 pM	Milk	[143]
Nanoconstructed lanthanum oxide nanoparticle-decorated reduced graphene oxide nanocomposite (nLa_2_O_3_ NP@rGO)-based platform functionalized with 3-aminopropyltriethoxysilane (APTES) and attachment on ITO-coated substrate. mAbs immobilized onto surface.	Ciprofloxacin	CV DPV EIS	0.055 μg mL^−1^	Milk	[144]
Tyramine (TA) electropolymerized resulting in an ultrathin polytyramine (PTA) film on a gold-coated silicon electrode (AuE) modified with polyclonal antibodies	Tetracycline	EIS	0.01 μg L^−1^	Spiked buffer	[145]

**Table 5:** Examples of gold-standard technologies for antibiotic detection, with analysis time and sample types.

Company	Technology	Product	LoD	Time Required	Sample Types	Ref
ThermoFisher Scientific	HPLC and MS	Vanquish UHPLC and Orbitrap MS	Ppb levels	30–60 min per sample	Food, water, biological fluids	[152]
Charm Sciences	Microbiological Assays	ROSA Lateral Flow System	Low ppb levels	1–2 h per batch	Food, dairy products	[153]
Sciex	CE	Capillary Electrophoresis System	Low ng/mL range	20–40 min per sample	Water, biological fluids, pharmaceuticals	[154]
Bruker Corporation (Billerica, MA, USA)	MS	MBT STAR-Carba IVD	Low ppb levels	10–20 min per sample	Food, water, pharmaceuticals	[155]
JEOL Ltd. (Tokyo, Japan)	NMR	ECZ500R NMR Spectrometer	Ppb levels	1–2 h per sample	Biological fluids, complex mixtures	[156]
Waters Corporation	LC-MS/UV–Vis	Xevo TQ-S micro LC-MS/MS System	Ppt levels	30–60 min per sample	Food, biological fluids, environmental samples	[157]

**Table 6:** Examples of immunoassay-based technologies for antibiotic detection, with analysis time and sample types.

Company	Product	Sample Types	Time Required	LoD	Ref
Meizheng (Rizhoa, China)	ELISA Kit	Food, water, biological tissues	1–2 h	0.05 ppb	[167]
Meizheng	Nitroimidazole ELISA Test Kit	Animal tissues and eggs	1–2 h	0.2 ppb	[168]
Creative Diagnostics (Shirley, NY, USA)	ELISA Kits for Drug Residues Detection	Water, food, Biological tissues	2 h	0.01–1.5 ppb	[169]
Neogen Corporation	Veratox for Tetracyclines	Dairy	30 min	1 ppb	[170]
Gold Standard Diagnostics (Warminster, PA, USA)	SENSISpec Tetracycline ELISA	Meat, milk, shrimp, and honey	1–2 h	0.05–2 ppb	[171]
Abcam (Cambridge, UK)	Antibiotic Residue Detection ELISA Kit	Tissues, Milk	1.5 h	0.1 ppb	[172]
MP Biomedicals (Irvine, CA, USA)	Quick Test Kit for Antibiotics	Milk, meat, seafood	1 h	0.2 ppb	[173]
BioVision (Zurich, Switzerland)	Antibiotic Residue ELISA Kit	Milk, Tissues	2 h	0.3 ppb	[174]

**Table 7:** Examples of electrochemical-based devices and sensors for the detection of antibiotics.

Company	Product	Sample Types	Time Required	LoD	Ref
Randox Food Diagnostics	Antibiotic Array	Milk, meat, fish	30 min	0.5 ppb	[175]
Charm Sciences Inc.	SLBL Beta-Lactam Test	Dairy products	~5 min	1 ppb	[176]
Metrohm (Bangkok, Thailand)	AN-P-037	Food, water	1 h	0.1 ppb	[177]
PalmSens	PalmSens4 Potentiostat	Food, environmental samples	~30 min	0.05 ppb	[178]
Dropsens (Metrohm)	Screen-Printed Electrodes	Food, water	~20 min	0.01–10 ppb	[179]
PalmSens	Sensit Smart	Food, clinical samples	~45 min	0.1 ppb	[180]
ZP (Zimmer and Peacock) (Skoppum, Norway)	ZP Anapot	Environmental samples	30 min	0.05 ppb	[181]
Analytik Jena (Jena, Germany)	Multi EA 5100	Water, food, biological samples	1 h	0.2 ppb	[182]
